# Rab27b Is a Potential Indicator for Lymph Node Metastasis and Unfavorable Prognosis in Lung Adenocarcinoma

**DOI:** 10.1155/2018/7293962

**Published:** 2018-12-02

**Authors:** Louqian Zhang, Weifei Fan, Li Xu, Qixing Mao, Yan Chen, Yuan Mao, Lin Xu, Jun Wang

**Affiliations:** ^1^Department of Thoracic Surgery, Jiangsu Cancer Hospital, Jiangsu Institute of Cancer Research, Nanjing Medical University Affiliated Cancer Hospital, Nanjing, China; ^2^The Fourth Clinical College of Nanjing Medical University, Nanjing, China; ^3^Jiangsu Key Laboratory of Molecular and Translational Cancer Research, Nanjing, China; ^4^Department of Hematology and Oncology, Department of Geriatric Lung Cancer Laboratory, Geriatric Hospital of Nanjing Medical University, Jiangsu Province Geriatric Hospital, Nanjing, China; ^5^Department of Pathology, Jiangsu Cancer Hospital, Nanjing, China

## Abstract

Rab27b is reported to associate with the development and progression of several types of human cancers. However, the relationship between Rab27b expression and the clinical characteristics of lung adenocarcinoma (LUAD) is rarely explored. In this present study, the TCGA database was consulted, followed by one-step quantitative reverse transcription polymerase chain reaction (qPCR), Western blot, and immunohistochemistry (IHC) analyses in LUAD cell lines and tissue samples. Rab27b expression levels were statistically higher in LUAD cell lines and tissue samples compared with a noncancerous cell line and tissue samples (*p* < 0.05). Rab27b expression was statistically correlated with lymph node metastasis (*p* = 0.016) and TNM stage (*p* = 0.019). Survival analysis and Kaplan-Meier curve revealed that Rab27b expression (*p* = 0.006) and TNM stage (*p* = 0.027) were independently associated with the unfavorable overall survival of patients with LUAD. These results indicate that high expression of Rab27b correlates with malignant attributes of LUAD and Rab27b may be identified as a potential indicator of metastasis and prognosis for LUAD.

## 1. Introduction

Lung cancer is currently the most common cause of cancer-related death worldwide [1]. In China, there are over 700,000 newly LC cases and almost 600,000 LC patients died annually [2]. Non-small-cell lung cancer (NSCLC) accounts for approximately 85% of all LC cases, and lung adenocarcinoma (LUAD) is the most common histologic subtype [3]. For LUAD therapy, the effectiveness of traditional therapeutic strategies, including surgery, radiation, and chemotherapy, is unsatisfactory for they failed to accomplish significant survival benefits [4]. Although tyrosine kinase inhibitors (TKIs) and immune checkpoint inhibitors have been widely used in recent years, the overall survival of LUAD patients is still frustrating. Identification of novel biomarkers with clinicopathological and prognostic significance is of great importance for LUAD management. Up to now, an increasing number of studies have detected the crucial molecular alterations of NSCLC and screened several promising biomarkers for LUAD [5, 6]. There continues a remarkable demand for the identification of potential molecular biomarkers, and alternative treatment strategies are offered for LUAD.

Rat sarcoma- (Ras-) associated binding (Rab) protein, which functions as molecular switches that alternate between active GTP-bound and inactive GDP-bound conformational states, exerts a significant role in endocytosis, exocytosis, and vesicle trafficking [7]. Rab27 is a special member of the small GTPase Rab family and composed of two isoforms, Rab27a and Rab27b [8]. Recently, mounting evidence has reported that elevated expression of Rab27b could be observed in various kinds of cancers and is highly associated with malignant behaviors [9, 10]. High Rab27b expression also indicated poor prognosis in ovarian cancer [11], breast cancer [12], pancreatic cancer [13], and colorectal cancer [14]. However, the role of Rab27b in LUAD remains unclear. The expression of Rab27b, as well as its clinical characteristics and prognostic significance in LUAD, deserves further investigation.

In this present study, we explored the Rab27b RNA expression in the TCGA database. Then, we detected the Rab27b expression in LUAD cell lines and tissue samples by using quantitative real-time polymerase chain reaction (qPCR) and Western blot tests. Moreover, we enrolled LUAD tissue microarray (TMA) to further examine Rab27b expression by immunohistochemistry (IHC) analysis. Finally, we analyzed the relationship between Rab27b expression and its clinicopathologic features in LUAD.

## 2. Materials and Methods

### 2.1. TCGA Database Consultation

The TCGA database was employed (https://cancergenome.nih.gov) to validate the RNA expression of Rab27b in LUAD tissues and corresponding noncancerous tissues.

### 2.2. Cell Lines

Three LUAD cell lines (H1299, H1975, and A549) and a human bronchial epithelial cell line (HBE) were obtained from the cell bank of the Chinese Academy of Science (Shanghai, China) and cultured routinely by our lab.

### 2.3. LUAD Sample Collection

Five fresh-frozen LUAD samples and corresponding noncancerous tissue samples were obtained from the Department of Thoracic Surgery, Nanjing Medical University Affiliated Cancer Hospital. Simultaneously, 80 cases of LUAD samples were enrolled to construct tissue microarrays (TMAs), and the TMAs were purchased from Outdo Biotech Co. Ltd. (Shanghai, China). The important clinical information, including gender, age, tumor size, pathological grade, histological type, tumor status (T), lymph node metastasis (N), distant metastasis (M), and TNM stage, was also provided from the original TMA data. None of the NSCLC patients received any forms of treatments (radiation therapy, chemotherapy, or immunotherapy) before surgery.

### 2.4. Ethics Statement

Written informed consent was obtained from each patient included in this study. Ethical approval to perform this research was approved by the Human Research Ethics Committee of Nanjing Medical University and each local hospital.

### 2.5. qPCR, Western Blot, and IHC Analyses

qPCR and Western blot analyses were performed in LUAD cell lines and LUAD samples. For the qPCR test, total RNA was extracted using Trizol (Invitrogen, USA) following the manufacturer's manual. The protocol was described previously [15]. The primers for Rab27b were as follows: forward primer 5′-TGC GGG ACA AGA GCG GTT CCG-3′ and reverse primer 5′-GCCAGT TCCCGAGCT TGCCGT T -3′. The glyceraldehyde 3-phosphate dehydrogenase (GAPDH) was used as an internal control, and the primers for GAPDH were as follows: forward primer 5′-TGC ACC ACC AAC TGC TTA GC-3′ and reverse primer 5′-GGC ATG GAC TGT GGT CAT GA-3′. For Western blot analysis, total protein from each lysate was loaded and separated by 10% SDS-PAGE and transferred onto the nitrocellulose membrane. The membranes were first incubated with a primary rabbit anti-Rab27b antibody (1 : 2000, ab229874, Abcam, Cambridge, MA, USA) and then a secondary antibody (DakoCytomation, Carpinteria, CA) and then were detected by the ECL kit and autoradiography using X-ray film. For IHC analysis, the detailed protocol was described previously [16, 17]. TMA sections were incubated with a primary rabbit anti-Rab27b antibody (1 : 100, ab133715, Abcam), and Rab27b immunostaining was evaluated according to the intensity and percentage of Rab27b-positive cells [14, 18–20]. Briefly, immunostaining intensity of Rab27b was categorized as follows: 0 (negative), 1 (weakly positive), 2 (moderately positive), and 3 (strongly positive). Immunostaining percentage of Rab27b was also scored as 4 levels, in which 1 was given for 0–10%, 2 for 11–50%, 3 for 51–80%, and 4 for 81–100%. The product of the intensity and percentage scores was employed as the ultimate immunohistochemistry score (IHS). The degree of Rab27b staining was determined using a two-level grading system as follows: <3 indicates low or no expression while ≥3 indicates high expression. The cut-off value of 90 was selected.

### 2.6. Statistical Analysis

All values were expressed as the mean ± standard error. The associations between Rab27b expression and clinicopathologic characteristics were calculated by chi-square tests. Univariate and multivariate Cox regression models were used to identify prognostic factors that affected the overall survival. *p* < 0.05 was considered to indicate a statistically significant difference. All statistical analyses were performed by utilizing STATA 14.0 (Stata Corporation, College Station, TX, USA) and SPSS 18.0 (SPSS Inc., Chicago, IL, USA).

## 3. Results

### 3.1. Rab27b Expression in the TCGA Database

**Figure 1:**
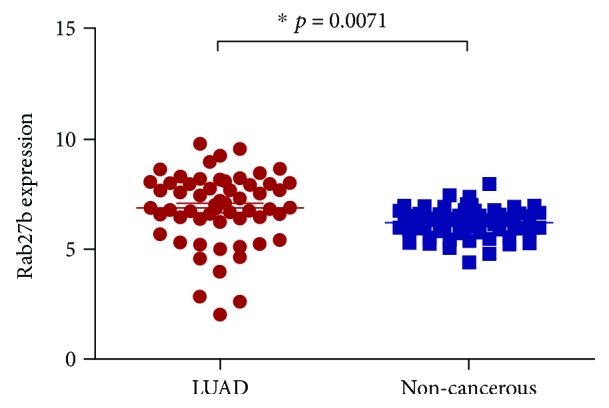
Rab27b expression in LUAD tissues is remarkably higher than that in corresponding noncancerous tissues (*p* = 0.0017) (TCGA database).

The TCGA database was employed, and 57 cases of LUAD were selected to preliminarily detect the RNA expression of Rab27b in LUAD tissues. As shown in Figure 1, Rab27b expression in LUAD tissues is remarkably higher than that in corresponding noncancerous tissues (*p* = 0.0017).

### 3.2. Rab27b Expression in LUAD Cell Lines and Tissue Samples

**Figure 2:**
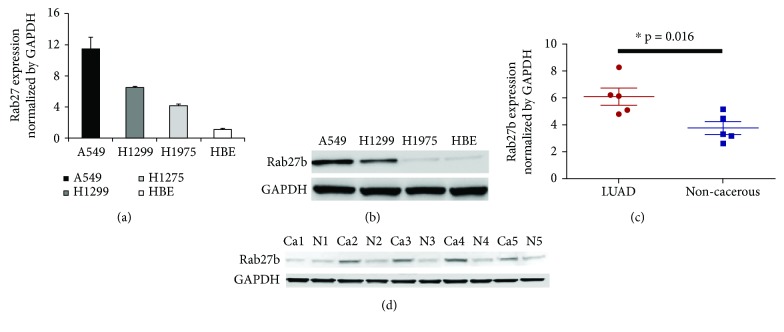
One-step quantitative real-time polymerase chain reaction (qPCR) test and Western blot analysis were performed to detect the mRNA and protein expression of Rab27b in lung adenocarcinoma (LUAD) cell lines and tissue samples. A549, H1299, and H1975 are three LUAD cell lines. HBE is a noncancerous human bronchial epithelial cell line. (a, b) qPCR and Western blot analyses demonstrated that Rab27b expression was significantly higher in LUAD cell lines than in a normal HBE cell line. (c, d) The expression of Rab27b was also elevated in LUAD tissues compared with noncancerous tissues. Ca: LUAD tissue samples; N: corresponding noncancer tissue samples.

qPCR and Western blot analyses were performed in LUAD cell lines and tissue samples to further investigate Rab27b expression. The results of both qPCR and Western blot analyses demonstrated that Rab27b expression was significantly higher in LUAD cell lines than in a normal HBE cell line (Figures 2(a) and 2(b)). Similarly, the expression of Rab27b was elevated in LUAD tissues compared with noncancerous tissues (Figures 2(c) and 2(d)).

### 3.3. Rab27b Expression in LUAD TMA

**Figure 3:**
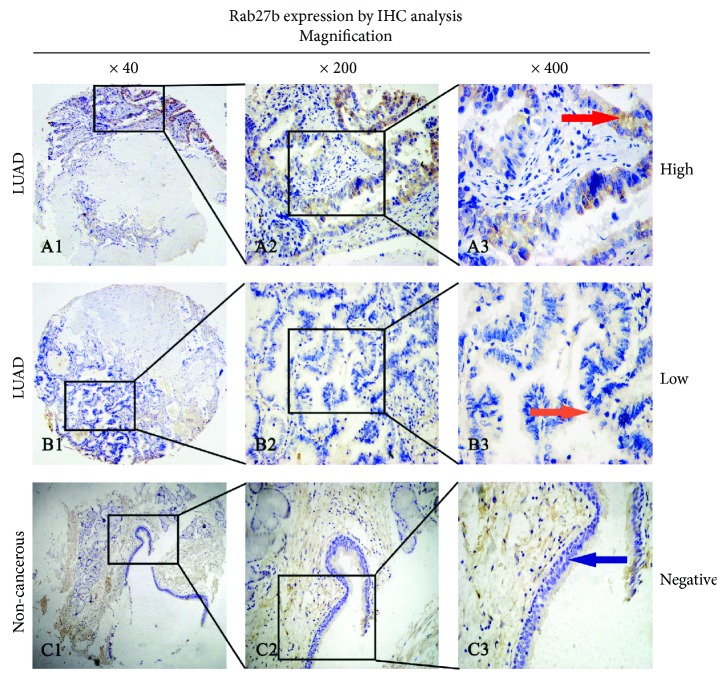
Representative types of Rab27b protein expression in lung adenocarcinoma (LUAD) tissue samples and corresponding noncancerous tissue samples. (A1, A2, and A3) High cytoplasmic expression of Rab27b in an LUAD tissue sample. Red arrows show the positive staining in the cytoplasm of cancer cells. (B1, B2, and B3) Low cytoplasmic expression of Rab27b in an LUAD tissue sample. Orange arrows show the positive staining in the cytoplasm of cancer cells. (C1, C2, and C3) No expression of Rab27b in a noncancerous tissue sample. Original magnification: ×40 in A1–C1, ×200 in A2–C2, and ×400 in A3–C3.

Positive IHC staining of Rab27b was mainly located in the cytoplasm of LUAD and normal tissues. High Rab27b cytoplasm expression was detected in 52 of 80 (65%) LUAD tissues compared with 36 of 80 (45%) noncancerous tissues, and the difference showed a remarkable significance (*χ*^2^ = 6.4646, *p* = 0.011). A few cases also showed nucleus expression of Rab27b (13 in LUAD tissues and 7 in noncancerous tissues). The IHC staining for Rab27b cytoplasm expression and its relationship with important clinical characteristics are illustrated in Figure 3 and Table 1. High Rab27b cytoplasm expression was significantly correlated with lymph node metastasis (*p* = 0.016) and TNM stage (*p* = 0.019).

### 3.4. Survival Analysis

**Figure 4:**
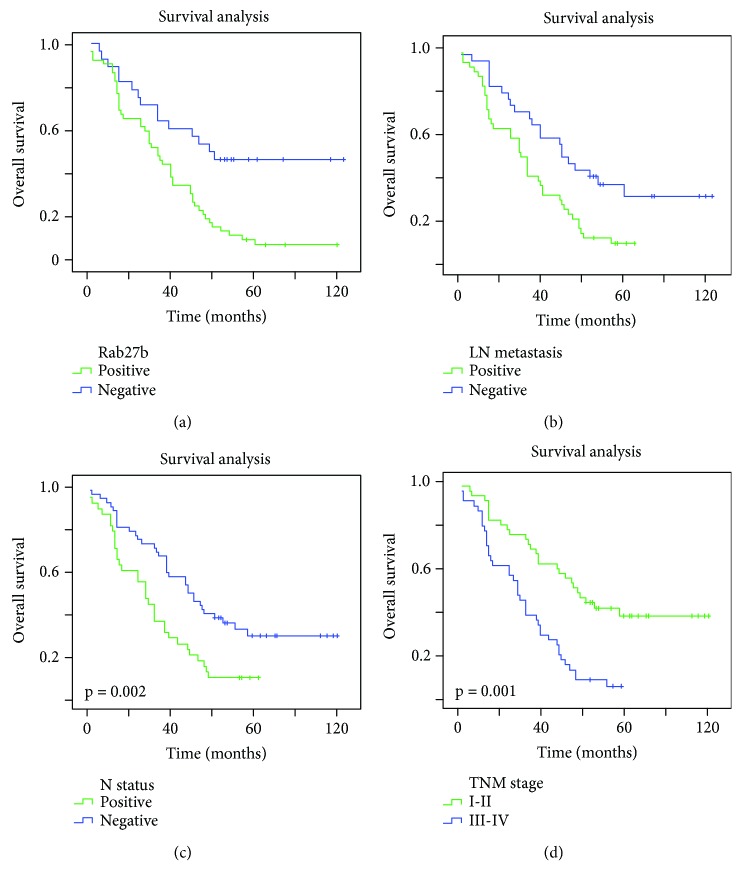
Survival analysis of lung adenocarcinoma (LUAD) patients by the Kaplan-Meier method. (a) The overall survival rate in patients with high Rab27b protein expression (green line) was significantly lower than that in patients with low Rab27b expression (blue line). (b) The overall survival rate in patients with positive lymph node metastasis (green line) was significantly lower than that in patients with negative lymph node metastasis (blue line). (c) The overall survival rate in patients with positive N status (green line) was significantly lower than that in patients with negative N status (blue line). (d) The overall survival rate in patients with advanced TNM stage (blue line) was significantly lower than that in patients with early TNM stage (green line).

The univariate analysis firstly screened four factors that were associated with the overall survival of 80 LUAD cases, including Rab27b expression (*p* = 0.002), lymph node metastasis (*p* = 0.007), N status (*p* = 0.014), and TNM stage (*p* = 0.001). Multivariate analysis further confirmed that Rab27b expression (*p* = 0.006) and TNM stage (*p* = 0.027) were two independent prognostic indicators for LUAD in this present study (Table 2). Kaplan-Meier survival curves were then built to indicate that NSCLC patients with high Rab27b expression and advanced T suffered unfavorable survival time (Figure 4).

## 4. Discussion

Rab proteins of both endocytic and exocytic pathways illustrate crucial functions in cancer progression [21, 22]. As a member of the secretory Rab27 subfamily, Rab27b showed rare expression in normal tissues while it showed high expression in several types of human cancers [23]. Although there were few mechanism researches that inspected the characteristics of Rab27b, it was identified to fuel invasion growth and metastasis of breast cancer and facilitate proliferation of HCC cells by regulating the PI3K/AKT/p21 pathway [9, 24]. All the above information implied the oncogenic characteristics of Rab27b. In this present study, we attempted to detect the differentiated expression of Rab27b in LUAD and evaluate the relationship between Rab27b expression and important clinicopathological features of LUAD, especially the prognosis significance.

The info of the TCGA database rudimentarily exemplified the Rab27b RNA expression in LUAD. Then, the mRNA and protein expression of Rab27b was investigated in LUAD cell lines. The data displayed that Rab27b expression was elevated in LUAD cells in comparison to noncancerous cells. Consistently, the results of qPCR and Western blot analyses showed that Rab27b expression in small LUAD samples was statistically upregulated than that in noncancerous tissues. IHC analysis in large LUAD cases further proved that the protein expression of Rab27b in LUAD TMA was also higher than that in noncancerous tissues. The above results concerning Rab27b expression were consistent with the data of previous researches that described the differentiated expression of Rab27b in several types of human cancers [11, 23]. Moreover, high Rab27b protein expression significantly correlated with important clinical attributes, including lymph node and TNM stage. The above data also agreed with the previous researches which stated the oncogenic behaviors of Rab27b in cancer development [9, 13].

In survival analysis, Rab27b expression, lymph node metastasis, N status, and TNM stage were screened as four elements that were significantly associated with the overall survival of LUAD patients by using a univariate mode. Multivariate analysis further identified that Rab27b and TNM stage may act as the independent indicators for LUAD prognosis. Kaplan-Meier curve also depicts that the lifespan of LUAD patients with high Rab27b expression suffered a poor outcome. These data agree with the previous studies that introduced the prognostic role of Rab27b in ovarian cancer [11], colorectal cancer [14], pancreatic cancer [23], breast cancer [12], hepatocellular carcinoma [24], and glioma [25].

However, Dong et al. reported that decreased expression of Rab27b correlated with metastasis and poor prognosis in colorectal cancer. Negative expression of Rab27b significantly correlated with tumor differentiation and positive vascular invasion. Moreover, positive expression of both Rab27a and Rab27b was a protective factor in CRC [26]. The presence of inconsistent data may be due to several reasons. For one thing, Dong et al. used Rab27a and Rab27b as a combined index, which could lead different evaluation criteria. For another, Rab27b may have multiplicate properties in different cancer types or under different circumstances. Future studies that enroll larger samples and convincible protocols are necessary.

There are two issues for this present study. Firstly, the study only analyzed OS data, not DFS (disease-free survival) or PFS (progression-free survival) data. The main reason is that the original TMAs only provide OS information. Secondly, the mechanism of Rab27b in LUAD was barely involved. We need to further explore how Rab27b affects the malignant behaviors of LUAD.

To sum up, this study reported the high expression of Rab27b in LUAD. Furthermore, the relationship between Rab27b expression and clinical characteristics of LUAD patients, especially prognosis condition, was investigated for the first time. Rab27b could be identified as a novel prognostic biomarker in LUAD.

## Figures and Tables

**Table 1:** Correlation of high Rab27b protein expression with clinicopathological characteristics in 80 LUAD cases.

Groups	No.	Rab27b (+)	*χ* ^2^	*p* value	
Gender				
Male	44	26	1.5007	0.221	
Female	36	26			
Age				
≥60 years	50	30	1.4652	0.226	
<60 years	30	22			
Tumor diameter				
≥3 cm	52	33	0.1546	0.694	
<3 cm	28	19			
Pathological grade				
Grades I–II	57	39	1.0199	0.313	
Grade III	23	13			
Lymph node metastasis				
Positive	46	35	5.8481	0.016^∗^	
Negative	34	17			
T status				
T1–T2	61	38	0.8260	0.363	
T3–T4	19	14			
N status				
Positive	35	26	2.3583	0.125	
Negative	45	26			
M status				
Positive	1	1	0.5453	0.460	
Negative	79	51			
TNM stage				
Stages I–II	40	21	5.4945	0.019^∗^	
Stages III–IV	40	31			

^∗^ *p* < 0.05; LUAD: lung adenocarcinoma.

**Table 2:** Univariate and multivariate analyses of prognostic factors for overall survival in 80 LUAD cases.

	Univariate analysis			Multivariate analysis		
	HR	*p* value	95% CI	HR	*p* value	95% CI
Rab27b expression						
High versus low	2.54	0.002^∗^	1.42–4.57	2.31	0.006^∗^	1.27–4.22
Gender						
Male versus female	1.19	0.497	0.72–1.96			
Age						
≥60 years versus <60 years	0.94	0.811	0.56–1.57			
Tumor diameter						
≥3 cm versus <3 cm	1.43	0.190	0.84–2.45			
Pathological grade						
Grades I–II versus grade III	0.91	0.728	0.52–1.57			
Lymph node metastasis						
Positive versus negative	2.07	0.007^∗^	1.23–3.50	0.74	0.507	0.30–1.81
T status						
T1–T2 versus T3–T4	0.70	0.212	0.39–1.23			
N status						
Positive versus negative	1.87	0.014^∗^	1.14–3.08	1.65	0.143	0.84–3.22
M status						
Positive versus negative	1.05	0.962	0.14–7.61			
TNM stage						
Stages I–II versus stages III–IV	0.43	0.001^∗^	0.25–0.71	0.45	0.027^∗^	0.22–0.91

^∗^ *p* < 0.05; HR: hazard ratio; CI: confidence interval; LUAD: lung adenocarcinoma.

## Data Availability

RNA expression of Rab27b in LUAD tissues and corresponding noncancerous tissues in the TCGA database could be downloaded in the following website: https://cancergenome.nih.gov. The original data of LUAD TMA could be downloaded in the following website: http://www.superchip.com.cn. All other data concerning this study could be provided by contacting the corresponding author: Dr. Jun Wang (wangjun1959@njmu.edu.cn, ORCID: 0000-0003-1797-2386).
